# TGFβ: Signaling Blockade for Cancer Immunotherapy

**DOI:** 10.1146/annurev-cancerbio-070620-103554

**Published:** 2021-12-22

**Authors:** Szu-Ying Chen, Ons Mamai, Rosemary J. Akhurst

**Affiliations:** 1 Helen Diller Family Comprehensive Cancer Center, University of California, San Francisco, California, USA; 2 Department of Anatomy, University of California, San Francisco, California, USA

**Keywords:** cancer, immunotherapy, TGFβ, integrins

## Abstract

Discovered over four decades ago, transforming growth factor β (TGFβ) is a potent pleiotropic cytokine that has context-dependent effects on most cell types. It acts as a tumor suppressor in some cancers and/or supports tumor progression and metastasis through its effects on the tumor stroma and immune microenvironment. In TGFβ-responsive tumors it can promote invasion and metastasis through epithelial-mesenchymal transformation, the appearance of cancer stem cell features, and resistance to many drug classes, including checkpoint blockade immunotherapies. Here we consider the biological activities of TGFβ action on different cells of relevance toward improving immunotherapy outcomes for patients, with a focus on the adaptive immune system. We discuss recent advances in the development of drugs that target the TGFβ signaling pathway in a tumor-specific or cell type–specific manner to improve the therapeutic window between response rates and adverse effects.

## 1. INTRODUCTION

Targeting the transforming growth factor β (TGFβ) signaling pathway for cancer therapy has repeatedly gone in and out of vogue. Recent renewed interest was spurred by clinical success of immunotherapies, particularly immune checkpoint blockade (ICB) agents (see the sidebar titled Immune Checkpoint Blockade), when placed in context with the potent immunosuppressive effects of TGFβ signaling in normal and tumor tissues (Arteaga et al. 1993, Gorelik & Flavell 2001, Thomas & Massague 2005, Flavell et al. 2010). The concept of blocking TGFβ signaling to potentiate ICB therapy was further stimulated by widespread findings that pretreatment human tumors refractory to anti-PD-1 (programmed cell death protein 1)/PD-L1 (programmed death-ligand 1) therapy are enriched for transcriptomic signatures of epithelial-mesenchymal transformation (EMT), extracellular matrix (ECM), immunosuppression, and TGFβ signaling, with TGFβ signaling probably driving the first three features (Hugo et al. 2016, Chakravarthy et al. 2018, Mariathasan et al. 2018, L. Wang et al. 2018, Desbois et al. 2020).

TGFβ (see the sidebar titled TGFβ Activities in Health and Disease) is produced by an array of tumor cell types, particularly myeloid-derived suppressor cells (MDSCs), cancer-associated fibroblasts (CAFs), tumor-associated macrophages (TAMs), and activated T cells, as well as malignant cells per se (Derynck et al. 2021). Moreover, in in vivo preclinical tumor models, anti-PD-1 therapy can exacerbate high TGFβ signaling (Dodagatta-Marri et al. 2019) through activation of another checkpoint on exuberant T cell activation, the secretion of TGFβ1 from activated CD4^+^ T helper type 1 (Th1) cells (Donkor et al. 2011, 2012), as well as other mechanisms.

Several drug companies are trialing TGFβ blockade in combination with anti-PD-1/PD-L1 therapies and other ICB therapies for advanced cancers, and there are recent extensive reviews on this topic (Dahmani & Delisle 2018, Batlle & Massague 2019, Groeneveldt et al. 2020, Derynck et al. 2021). We do not attempt to duplicate this effort here; instead we provide a brief introduction to the TGFβ signaling pathway and update new results and directions recently published, for example, using drugs targeting the activation of TGFβ or those that target TGFβ signaling in specific cell types or locations.

## 2. TGFβ INTRACELLULAR SIGNALING

**Figure 1:**
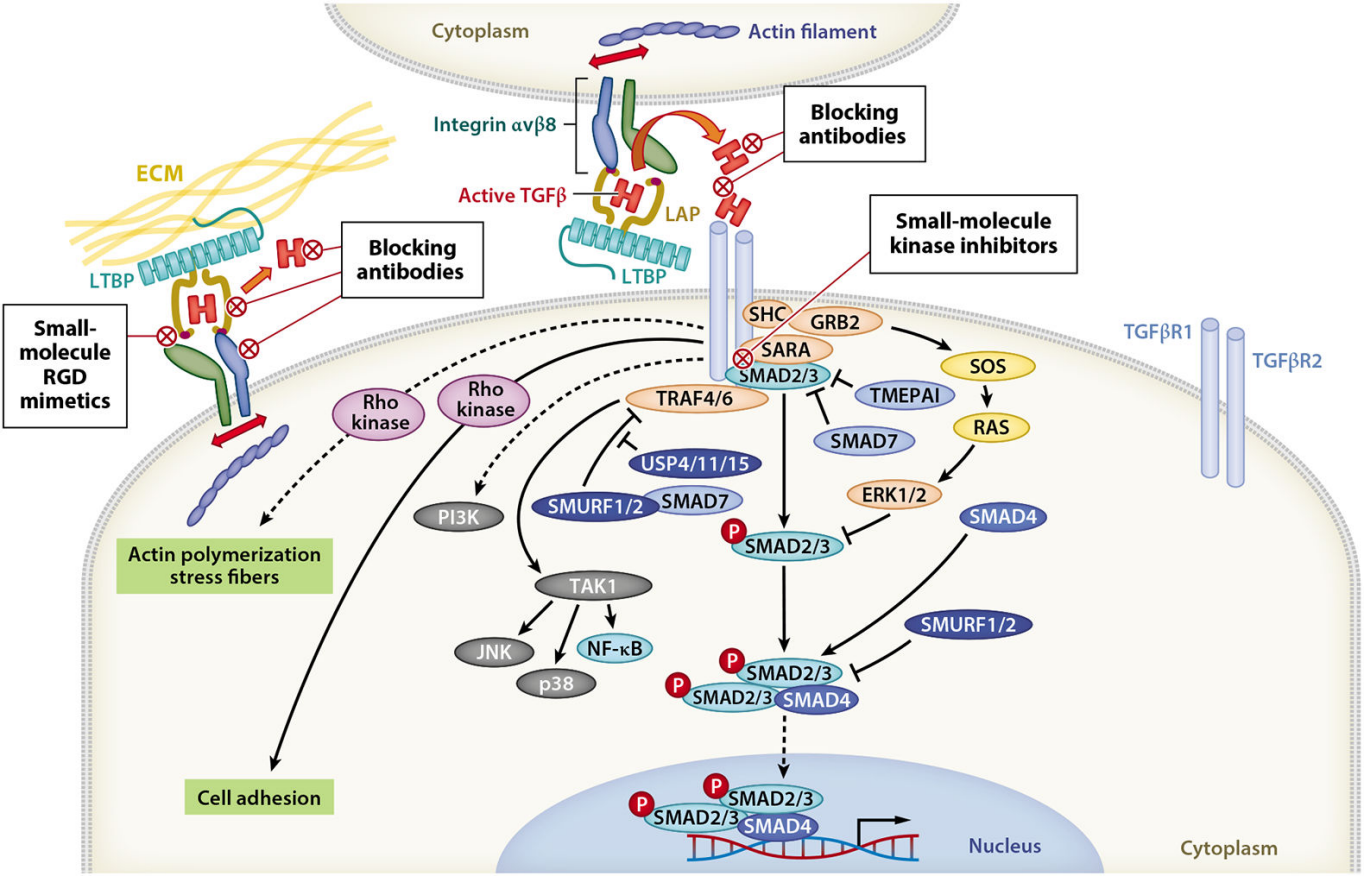
TGFβ signaling pathway showing druggable targets. The TGFβ receptors are dual-specificity kinases capable of phosphorylating serine/threonine and tyrosine residues. The canonical SMAD signaling pathway requires ligand-induced kinase activity of TGFβR2, which phosphorylates TGFβR1. TGFβR1 then phosphorylates the receptor-associated SMADs, SMAD2 and SMAD3. Phosphorylated SMAD2/3 forms a hexameric complex with SMAD4 and shuttles to the nucleus to initiate transcriptional responses that are context dependent and influenced by the availability of other transcription factors and cofactors. The TGFβ receptors can also directly activate other non-SMAD signaling pathways, including PI3K/AKT/mTORC, JNK, p38 MAPK, MEK/ERK, NF-κB/JAK/STAT, and Rho kinases. These pathways are activated by TGFβ binding to its receptors within distinct subcellular compartments (caveolae versus clathrin-coated pits), often with slower kinetics, and with lower magnitude of signal transduction than their activation by other stimuli. Non-SMAD and SMAD signaling pathways compete; for example, several non-SMAD pathways require SHCA binding to TGFβR1, and SHCA competes with R-SMADs for binding to TGFβR1. SARA potentiates R-SMAD binding to TGFβR1, while SMAD7 and TMEPAI antagonize this binding. Whereas the SHCA/GRB2/RAS/ERK pathway depends on TGFβR1 kinase activity, TRAF4/6 mediates ligand-activated signaling of JNK, p38 MAPK, and NF-κB pathways independent of TGFβR1 kinase activity. In this case, TGFβ induces recruitment of TAK1 to the type I receptor by its association with TRAF4 or TRAF6, which are RING domain E3 ubiquitin kinases. TRAF4/6 is activated by ligand-induced conformational changes in TGFβR1, causing ubiquitination and consequent activation of this kinase and its downstream pathways. Ubiquitination of TRAF4/6 and of SMADs by SMURF1/2 results in degradation of these targets, with USP deubiquitinases counteracting this activity. Abbreviations: ECM, extracellular matrix; LAP, latency-associated peptide; LTBP, latent TGFβ-binding protein; RGD, arginylglycylaspartic acid; TGFβR1, TGFβ receptor type 1; TGFβR2, TGFβ receptor type 2.

Mature bioactive TGFβ is a disulphide-linked protein of two 112–amino acid monomers. Each TGFβ isoform, TGFβ1, -β2, and -β3, activates intracellular signaling by binding to the transmembrane TGFβ type 2 receptor kinase (TGFβR2). This initiates the formation of a complex with the TGFβ type 1 receptor kinase (TGFβR1) to trigger a kinase cascade culminating in serine phosphorylation of the TGFβ receptor–associated SMADs (R-SMADs), SMAD2 and/or SMAD3. Phospho-R-SMADs bind SMAD4, forming the hexameric SMAD complex [R-SMAD2/3]_4_[SMAD4]_2_ that shuttles to the nucleus, interacts with other transcription factors and cofactors, and elicits context-dependent transcriptional responses (Figure 1).

Intriguingly, the same R-SMADs, SMAD2 and SMAD3, are utilized in signaling from activins, inhibins, Nodal, myostatin, and several growth and differentiation factors, which is a consideration when utilizing small-molecule inhibitors (SMIs) of SMAD2/3 phosphorylation, such as galunisertib or vactosertib. When large molecules are used as drugs, another consideration is drug access since TGFβR2/1 SMAD2/3 signaling is not executed at the plasmalemmal cell surface, but rather within endosomes following clathrin-mediated endocytosis of the ligand/receptor complex (Ehrlich et al. 2001, Penheiter et al. 2002, Di Guglielmo et al. 2003), and since latent TGFβ activation and signaling are tightly linked (Campbell et al. 2020, Seed et al. 2021). Conversely, translocation of intact TGFβR1 from recycling endosomes to the plasmalemma can be rapidly initiated by several stimuli, including exposure to glucose or insulin, promoting sensitization to ligand binding (Budi et al. 2015). TGFβ-TGFβR2/1 can also signal via noncanonical non-SMAD pathways, including TRAF6, PI3K, ERK, Akt, and mTOR, some of which are activated by TGFβ receptors within caveolae (Derynck & Budi 2019). These may also be targeted pharmacologically but are not considered TGFβ-specific pathways and are not discussed here (Figure 1).

## 3. ACTIVATION OF TGFβ: A TARGET IN DRUG DEVELOPMENT

**Figure 2:**
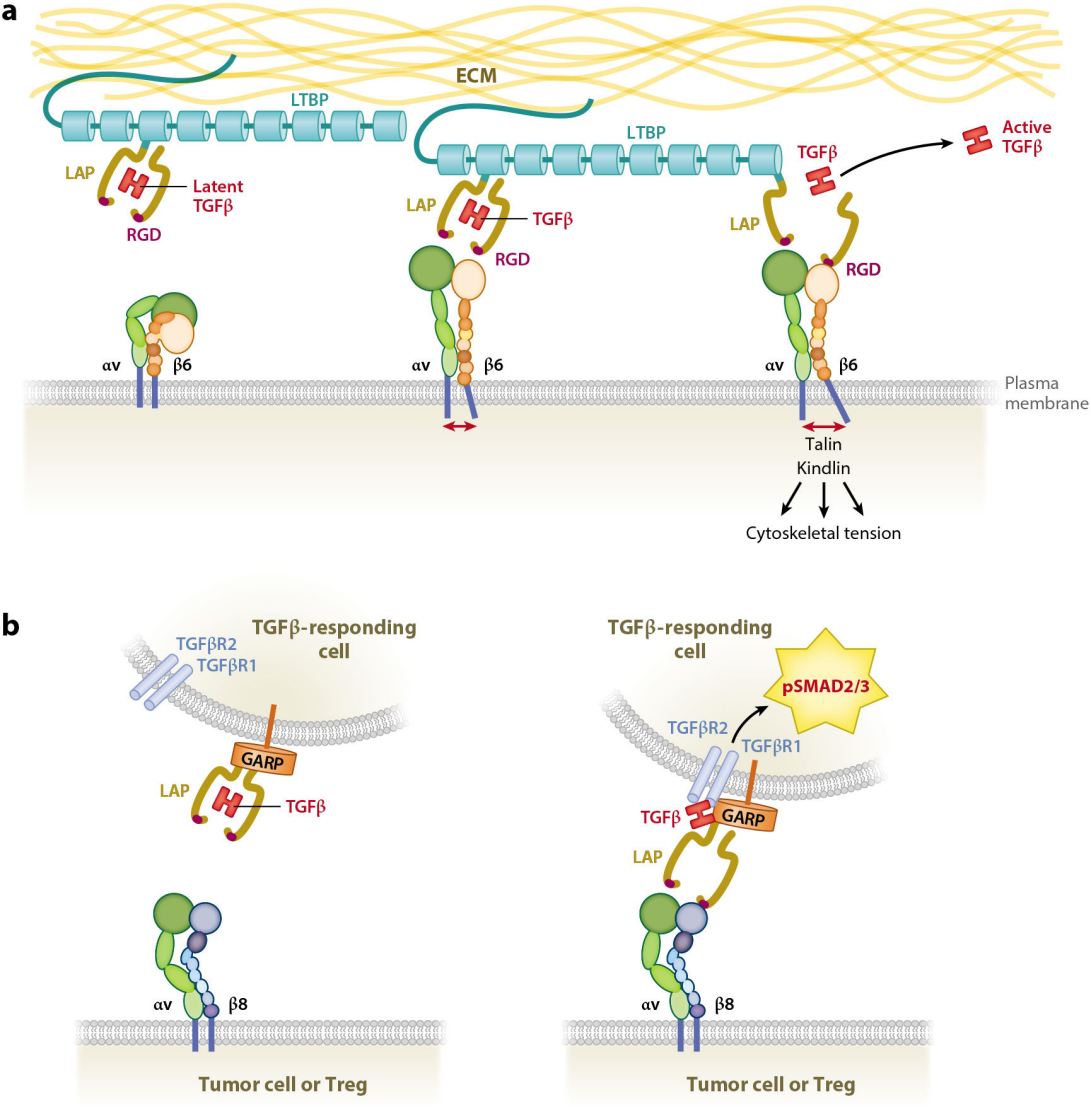
Activation of latent TGFβ. (*a*) αvβ6-mediated activation of the large latent complex of TGFβ tethered to the ECM via LTBP and released into the milieu or onto the surface of a responding cell. (*b*) αvβ8-mediated activation of a GARP-tethered latent complex on the surface of a T cell (as an example), without complete release of active TGFβ from the latent complex. This does not exclude the possible release of TGFβ from the latent complex after activation by integrin β8. The RGD site that contacts β integrins is shown in magenta within the yellow LAP. Abbreviations: ECM, extracellular matrix; GARP, glycoprotein A repetitions predominant; LAP, latency-associated peptide; LTBP, latent TGFβ-binding protein; RGD, arginylglycylaspartic acid; TGFβR1, TGFβ receptor type 1; TGFβR2, TGFβ receptor type 2; Treg, regulatory T cell.

The TGFβ ligands are synthesized as large precursor proteins, and the release of mature bioactive TGFβ is a major gateway for the regulation of TGFβ bioavailability (Figure 2). The molecular details of this process have received considerable attention in recent years due to the quest to find molecules that block TGFβ activation for therapeutic purposes. The primary translation product of TGFβ1 is 390 amino acids, with a short signal peptide followed by a long latency-associated peptide (LAP) and C-terminal bioactive ligand. During processing in the Golgi apparatus, the ligand is cleaved from its amino terminal LAP by furin but remains noncovalently associated within a taut cage composed of dimeric LAP molecules. This latent complex can be stabilized by a disulphide bond between a LAP and a LTBP (latent TGFβ-binding protein), which is a component of the ECM (Robertson & Rifkin 2016). Alternatively, it can bind covalently or noncovalently with other ECM or transmembrane milieu proteins, such as GARP (glycoprotein A repetitions predominant), also known as LRRC32 (leucine-rich repeats–containing protein 32), on regulatory T cells (Tregs) (Wang et al. 2012) or NRROS (negative regulator of reactive oxygen species; also known as LRRC33) on microglia (Qin et al. 2018).

Latent TGFβ can be activated in vitro by heat, low pH, or irradiation, as well as by nonspecific proteases, but β integrins complexed with integrin αv, particularly αvβ6 and αvβ8, are major physiological activators of latent TGFβ in vivo (Munger et al. 1998, 1999; Mu et al. 2002; Ludbrook et al. 2003) (see the sidebar titled Integrins). The LAPs for TGFβ1 and TGFβ3 possess Arg-Gly-Asp (RGD)-containing motifs exposed on the outer side of the LAP cage, and through ligation to αvβ6 or αvβ8 integrins, the bioactive TGFβ dimer is released or exposed, making it available for binding and signaling through the TGFβ receptor complex (Figure 2). TGFβ2 LAP does not possess an RGD site, and whether TGFβ2 activation is integrin dependent or independent is unknown.

The interaction of integrins with TGFβ LAPs occurs mainly through heterotypic cell-cell interactions between an integrin expressed on one cell type binding and activating latent TGFβ synthesized and secreted by another (Sato et al. 1990, Arnold et al. 2012, Nolte & Margadant 2020). However, latent TGFβ may also be activated by integrins coexpressed on the same cell type, particularly on immune cells (Edwards et al. 2014, Stockis et al. 2017). Elegant X-ray crystallography studies have revealed the mechanism of activation of TGFβ by integrin αvβ6, whereby ligation of a LAP to αvβ6, while tethered to a milieu protein, leads to major conformational changes in both the LAP and integrin (Shi et al. 2011, Dong et al. 2014). Conformational changes in integrin result in outside-in signaling to generate cytoskeletal tension through interaction with talins and microfilaments, while extracellular tension generated by integrin binding to RGD sites in the LAP results in the opening of the latency cage and release of mature bioactive TGFβ (Figure 2a).

Integrin αvβ8 is distinct from other αv-associated β integrins since it binds specifically to RGDLXXI/L or RGDLXXL/I motifs that are found only within TGFβ LAPs, in contrast to other more promiscuous integrins. Integrin β8 therefore has unique specificity for activation of TGFβ1 and TGFβ3 (Ozawa et al. 2016, Campbell et al. 2020). Integrin β8 is also distinguished by its truncated cytoplasmic tail, which some researchers predict cannot instigate outside-in signaling due to lack of an NPxY motif required for binding cytoskeletal signaling molecules like RABGAP1 and talin (Campbell et al. 2020), although other researchers have reported that integrin β8 may possess intracellular signaling capabilities (Reyes et al. 2013, Guerrero et al. 2017). Finally, integrin β8 shows more restrictive expression than other αvβ integrins and is upregulated within tumors compared to normal tissue, where it is expressed primarily on CD4^+^ T cells and malignant cells (Guerrero et al. 2017, Dodagatta-Marri et al. 2021, Laine et al. 2021, Seed et al. 2021).

Recent studies have revealed further molecular distinctions between the mechanisms of TGFβ activation by αvβ6 versus αvβ8. Uniquely, integrin β8 is constitutively structured in an extended conformation, and ligation of αvβ8 integrin to latent TGFβ presented on the cell surface by GARP causes flexibility in the LAP to expose the mature TGFβ ligand, making it available for TGFβ receptor engagement and signaling while still physically attached to the LAP (Figure 2b). Moreover, LAP binding does not cause a conformational change in β8 integrin, supporting the concept that integrin β8 ligation to the LAP does not trigger outside-in signaling (Campbell et al. 2020).

## 4. HETEROGENEOUS MECHANISMS DRIVE TUMOR RESPONSES TO ANTI-TGFβ IMMUNOTHERAPY

Tumors are complex and dynamic tissues, composed of many cell types and distinct ECMs. Apart from tumor cells per se, which are in a constant state of genetic, genomic, and epigenetic evolution, the tumor is composed of CAFs, smooth muscle cells, abundant ECM, blood and lymphatic vessels, and a quantitively and qualitatively diverse population of tumor-infiltrating leucocytes. Invasive tumors also incorporate host tissue, including bone, muscle, and adipose, which in turn can influence the biology of the tumor and its accessibility to anticancer drugs and immune cells. These heterogeneous cell types evolve together, influenced by cell type–specific secretion of cytokines, chemokines, and ECM molecules that constantly remodel the tumor.

Cancers develop from distinct cells of origin and display vastly differing cellular, genetic, and molecular phenotypes, such that each tumor is unique. Pancreatic cancers, often driven by mutant KRas and, commonly, genetic loss of *SMAD4,* have an extraordinarily high content of stromal fibroblasts and ECM interspersed with pockets of thriving tumor cells. This deadly cancer has a low tumor mutation load (TML), few infiltrated immune cells, and is refractory to most treatments. ER^+^ or HER2^+^ breast cancers have low mutational burden, low PD-L1 expression, defective antigen processing/presentation, and an immunosuppressive tumor microenvironment (TME), making them refractile to ICB. These tumor types contrast with squamous cell carcinomas (SCCs), which have a high tumor cell content, high TML, fewer CAFs, and less ECM, but greater immune cell infiltration.

Since TGFβ has pleiotropic activity on most cell types of the tumor and TGFβ blockade is not cytotoxic, it comes as no surprise that the mechanisms of action of anti-TGFβ therapy are as variable as the range of tumor types observed in the clinic. The question is, how does one capitalize on TGFβ blockade drugs using informed choices of drug combinations for each tumor type, optimal drug dosing regimens, and biomarker-guided therapy? For this, a deeper understanding of the mechanisms of action of TGFβ signaling and its inhibition in tumor growth and regression will be required for each tumor type.

## 5. IMMUNE MECHANISMS OF TGFβ BLOCKADE

### 5.1. TGFβ Signaling Effects on Innate Immune Cells of the Tumor

Rapid defense against foreign particles, whether viral, bacterial, or tumor cells, depends on the innate immune system. Myeloid, epithelial, and fibroblast cells are activated by the presence of danger-associated molecular patterns (DAMPs) that engage with transmembrane Toll-like receptors, cytoplasmically located NOD-like receptors, C-type lectin receptors, or AIM2 to trigger inflammation. DAMPS may be proteins, lipids, carbohydrates, nucleic acids, or ATP released by stressed or dying cells.

Myeloid cells of the innate immune system are the first responders in inflammation and can have cytotoxic [e.g., natural killer (NK) cells] or phagocytic [e.g., macrophages or dendritic cells (DCs)] abilities. Professional antigen-presenting cells, such as DCs and some macrophages, migrate to peripheral lymph nodes and prime naïve T cells for an antigen-specific adaptive immune response driven by T lymphoctyes.

Mechanisms have evolved to limit collateral damage to normal tissue caused by the release of inflammatory reactive oxygen species and free radicals, and these include secretion of TGFβ. However, excessive TGFβ within the tumor produces a state of chronic ineffectual inflammation (a wound that will not heal). This cytokine dampens the cytotoxic effects of NK cells, attracts monocytes and macrophages, and skews their differentiation from a type I differentiated cell state toward a type 2 regenerative phenotype that synthesizes yet more TGFβ and other immunosuppressive cytokines. The molecular and cellular details of TGFβ activities on innate immune cells are beyond the scope of this review but have been recently detailed by others (Batlle & Massague 2019, Derynck et al. 2021). In brief, in suppressing innate immune cells, TGFβ action hijacks their function toward a protumorigenic immune-suppressive role that is critical in dampening adaptive T cell–mediated antitumor immune responses. However, ultimately it is adaptive T cell–mediated immunity that is critical for the elimination of tumors and the establishment of long-term immunity.

### 5.2. Activation and Recruitment of CD8^+^ T Cells to the Tumor

Naïve T cells are activated in an antigen-specific manner within tumor-draining lymph nodes. DCs traffic between the tumor and lymph node, phagocytosing tumor cell debris and presenting peptide antigens, which are displayed on MHC-I (major histocompatibility complex class I) and MHC-II molecules, to T cell receptors (TCRs) on naïve CD8^+^ and CD4^+^ T cells, respectively. ICB therapies (see the sidebar titled Immune Checkpoint Blockade) are most effective when tumors have a high TML induced by environmental damage (chemical, sun-induced, diet-induced), such as with non-small-cell lung carcinoma (NSCLC), melanoma, and head and neck SCC (Schumacher & Schreiber 2015, Van Allen et al. 2015, Danilova et al. 2016, Hugo et al. 2016, Thorsson et al. 2018). This is likely due to the numerical increase in tumor-specific peptide neoantigens that increase the chance of a productive antitumor cytotoxic T cell response. Murine tumors also exhibit this relationship between TML and ICB response, and anti-TGFβ therapy is influenced by TML. In a panel of six independent chemically induced SCCs, all driven by mutant KRas and derived from the same FVB inbred mouse strain, only two with the highest nonsynonymous single-nucleotide TML showed tumor growth inhibition (TGI) to anti-TGFβ, anti-PD-1 monotherapy, or a combination thereof, whereas lower-TML tumors were unresponsive (Dodagatta-Marri et al. 2019).

It is widely accepted that TGFβ has direct immunosuppressive activity on CD8^+^ cytotoxic T cells, the critical warheads that eliminate tumor cells through antigen-specific synaptic engagement and injection of cytotoxic enzymes. TGFβ is a potent inhibitor of CD8^+^ T cell proliferation through the suppressive binding of transcription factors SMAD2/3 and ATF1/CREB to gene promoters encoding the cytotoxic arsenal of the CD8^+^ T cell, granzyme B (*GZMB*), perforin (*PRF1*), and IFNγ (*IFNG*) (Thomas & Massague 2005). TGFβ raises the threshold of antigen-TCR binding required for TCR signaling in both CD8^+^ and CD4^+^ T cells; consequently, TGFβ inhibition lowers this threshold, allowing for TCR stimulation by weaker antigens (Zhang & Bevan 2012). This likely plays an important role in tumor rejection by the TGFβ signaling blockade (Gunderson et al. 2020) and may be responsible for the phenomenon of antigen spread (Gulley et al. 2017) observed in response to combinatorial treatment of 4T1 mammary carcinomas treated with galunisertib and anti-PD-L1 (Holmgaard et al. 2018). In MC38 and CT26 mouse colon cancer models, *Tgfbr1* knockout in CD8α^+^ cells released T cells from the immunosuppressive activity of TGFβ (Gunderson et al. 2020), whereas deletion of *Tgfbr2* had no effect on tumor outgrowth in either MC38 or MMTV-PyMT mouse mammary tumor models (Li et al. 2020), possibly highlighting a difference between TGFβR1 and TGFBR2 signaling blockade in CD8^+^ T cells.

More recently it has become clear that TGFβ signaling blockade by galunisertib or anti-TGFβ antibodies not only increases the number and differentiation status of cytotoxic T cells within the tumor but also stimulates the migration of T cells from tumor stroma into the parenchyma of immune-excluded tumors, with consequent tumor regression or rejection (Mariathasan et al. 2018, Tauriello et al. 2018, Dodagatta-Marri et al. 2019, Desbois et al. 2020, Gunderson et al. 2020). Several mechanisms have been proposed. Transcriptomic analysis has highlighted the role of TGFβ-activated CAFs in immune exclusion of urothelial and ovarian cancer, which may mediate T cell exclusion by synthesizing a dense ECM physical barrier (Mariathasan et al. 2018, Desbois et al. 2020). TGFβ-activated CAFs also synthesize immunosuppressive cytokines, including TGFβ per se, as well as IL-6, IL-11, and TNFAIP6 (Desbois et al. 2020). TGFβ-mediated downregulation of MHC-I on tumor cells by TGFβ (David et al. 2017, Dodagatta-Marri et al. 2019, Desbois et al. 2020) may contribute to immune exclusion, although whether this phenomenon affects the structural organization of the tumor remains to be shown.

Direct inhibition of CD8^+^ T cell trafficking by TGFβ was demonstrated in the MC38 and CT26 colon cancer models, whereby administration of the TGFβR1 kinase inhibitor galunisertib prior to therapy enhanced TGI due to 5-fluorouracil plus radiation. Genetic deletion of *Tgfbr1* in Tregs or macrophages did not affect this outcome, whereas conditional deletion of *Tgfbr1* using a CD8α gene promoter dramatically ablated response to therapy (Gunderson et al. 2020). In addition to confirming that TGFβ suppresses differentiation and growth of CD8^+^ T cells, this study showed that *Tgfbr1*-deficient CD8^+^ T cells exhibit increased CXCR3 expression because of loss of pSMAD2 binding to the *Cxcr3* gene promoter, where it acts as a transcriptional suppressor. This upregulation of CXCR3 on CD8^+^ T cells enhanced their migration toward the IFNγ-inducible ligands CXCL9, -10, and -11 to stimulate trafficking into the tumor (Gunderson et al. 2020), and presumably also increased trafficking of CD4^+^ T and NK cells, which are also regulated by this mechanism (Nagarsheth et al. 2017). Importantly, in human colorectal cancer, CXCL10 was found to be associated with granzyme B–expressing CD8^+^ T cell infiltration and more favorable tumor, nodes, and metastases staging (Zumwalt et al. 2015). The authors of this study concluded that CD8α^+^ T cells are the direct target of TGFβ inhibitors in this model. Nevertheless, CD4^+^ T cell involvement was not ruled out since depletion of CD4^+^ T cells was redundant with the effect of galunisertib on chemotherapy and radiation therapy, suggesting that CD4^+^ T cells are the source of TGFβ immunosuppression of CD8^+^ cytotoxic T cells (Gunderson et al. 2020).

High-dose radiation can stimulate the CXCR3 chemokines CXCL9, -10, and -11 through upregulation or type 1 and type 2 interferons (Muroyama et al. 2017). Therefore, radiation and TGFβ inhibition may synergize by enhancing T cell trafficking into the tumor through upregulation of both CXCR3 and its ligands. Radiation also causes cell death and DNA damage, leading to the release of DAMPs and the generation of new antigens. With TGFβ blockade lowering the threshold for TCR activation, this results in a highly efficacious antitumor response (Formenti et al. 2018, Rodríguez-Ruiz et al. 2019).

### 5.3. CD4^+^ T Cells and TGFβ Signaling Blockade

**Figure 3:**
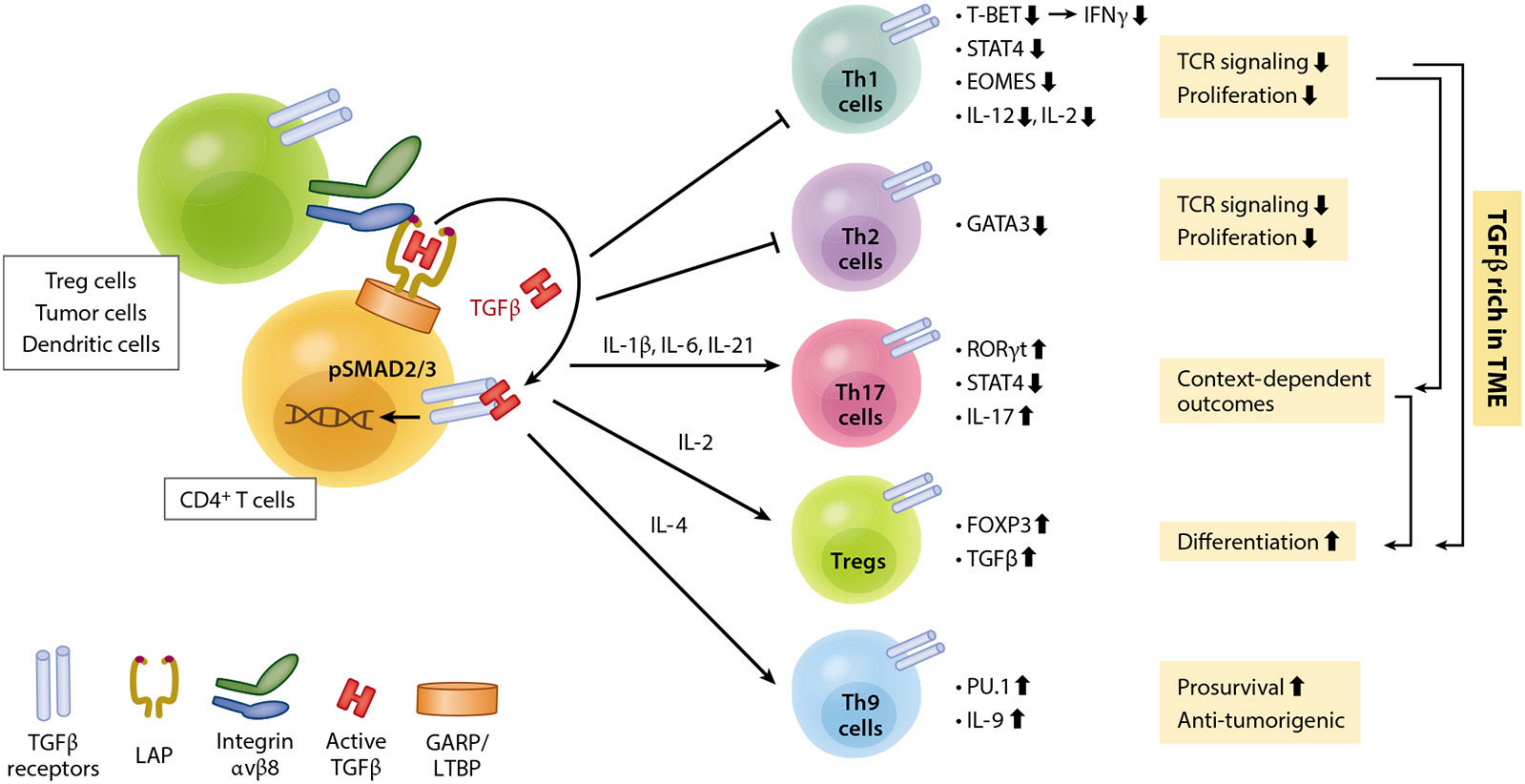
TGFβ is a master regulator of CD4^+^ T cell plasticity and function. Schematic of context-dependent TGFβ-mediated CD4^+^ T cell lineage choices that are determined by the presence of other cytokines in the milieu. Abbreviations: GARP, glycoprotein A repetitions predominant; LAP, latency-associated peptide; LTBP, latent TGFβ-binding protein; TCR, T cell receptor; Th, T helper; TME, tumor microenvironment; Treg, regulatory T cell.

CD8^+^ T cell activity within tumors is positively and negatively regulated by many cell types, including myeloid immune cells and CAFs. Most importantly, CD4^+^ T cells are the professional regulators of CD8^+^ T cell activity. These are an abundant but heterogeneous and dynamic T cell population in the tumor, and TGFβ is a major regulator of CD4^+^ T cell plasticity (Figure 3). The outcomes of TGFβ action on CD4^+^ T cells are context dependent, influenced by the presence or absence of other cytokines that determine differentiation along distinct CD4^+^ T cell lineages (Figure 3). In general, TGFβ suppresses activation and proliferation of naïve CD4^+^ T cells and of effector CD4^+^ Th1 and Th2 cells, while potentiating differentiation and proliferation along the immunosuppressive Treg lineage, although TGFβ signaling is not subsequently required for the function or maintenance of mature FOXP3^+^ Tregs (Ishigame et al. 2013b, Konkel et al. 2017, Gunderson et al. 2020, Li et al. 2020). Recent studies have found that differentiated CD4^+^ Tregs retain some degree of plasticity with respect to cell fate changes (McClymont et al. 2011), particularly in the setting of a tumor (D. Wang et al. 2018).

A common theme of several recent preclinical therapeutic studies is that high CD4^+^ T cell levels may be predictive of a favorable response to TGFβ signaling blockade, potentiating TGI and immune-mediated tumor regression in response to ICB (Dodagatta-Marri et al. 2019, Jiao et al. 2019, Li et al. 2020, Liu et al. 2020). However, the devil is in the details, with several mechanisms proposed by different studies dependent on the tumor type and stage.

### 5.4. Aggressive Cytotoxic CD8^+^ and CD4^+^ T Cells Induced by TGFβ Signaling Inhibition

Targeted inhibition of TGFβ signaling in T cells by genetic deletion or expression of a dominant negative TGFβR2 can lead to de novo acquisition of aggressive NK cell–like features, including expression of NKGD2, FasL, perforin, granzymes, and IFNγ on CD4^+^ (and CD8^+^) T cells (Marie et al. 2006, Ishigame et al. 2013a). The appearance of aggressive human CD8^+^ NK-like cells has been observed in response to bintrafusp alfa, a bispecific anti-PD-L1-TGFβ trap, when used in human tumor models xenografted into humanized NSG-β2m^−/−^ mice (Morillon et al. 2020). CD4^+^ T cells that express NK cell markers and cytotoxic enzymes have also been reported in human bladder cancer (Oh et al. 2020), NSCLC (Guo et al. 2018), melanoma (Kitano et al. 2013), and hepatocellular carcinoma (Zheng et al. 2017), and these CD4^+^ NK-like T cells have been shown to have tumor killing activity (Oh et al. 2020). It is therefore likely that TGFβ blockade will cause expansion or de novo differentiation of these cytotoxic CD4^+^ T cells, thereby contributing to tumor cell killing.

### 5.5. Synergy Between Anti-PD-1 and Anti-TGFβ Mediated Through CD4^+^ Regulatory T Cells

In most syngeneic mouse studies, anti-PD-1 therapy has little effect on tumor outgrowth when used as monotherapy (Curran et al. 2010). In mouse models of chemically induced high-TML SCC, Tregs are the most common CD4^+^ T cell type and they express high levels of PD-1. PD-1 blockade therefore prevents exhaustion not only of cytotoxic CD8^+^ T cells, but also of immunosuppressive CD4^+^ Tregs. The resultant competing immunosuppressive program reduces CD8^+^ T cell/Treg and CD4^+^ Th1 cell/Treg ratios, elevates TGFβ signaling, and enhances tumor cell EMT (Dodagatta-Marri et al. 2019), all features that have been associated with poor survival following ICB therapy in the clinic (Baras et al. 2016, Hugo et al. 2016, Mariathasan et al. 2018, Huang et al. 2019).

Similar observations have been made in clinical trials of anti-PD-1 therapeutics. Hyperprogression of gastric cancer observed in some patients after PD-1 blockade was attributed to activation and expansion of PD-1^+^ Tregs (Kamada et al. 2019), and in a trial of grade III/IV melanoma, high-PD-1^+^ Treg expansion was associated with melanoma recurrence and poor disease-free survival (Huang et al. 2019). Similarly, anti-CTLA4 expands both the CD4^+^ effector and CD4^+^ FOXP3^+^ Treg populations, with the latter being more sensitive to lower doses of anti-CTLA4 (Kavanagh et al. 2008, Jiao et al. 2019). Notably, since Tregs are induced in response to TGFβ (Chen et al. 2003) and present and activate latent TGFβ through GARP/integrin β8 (Tran et al. 2009, Stockis et al. 2017), and since the *TGFB1* gene is auto-inductive (Yue & Mulder 2000), this creates a positive feedforward TGFβ signaling circuit that drives immunosuppressive protumorigenic Treg differentiation after PD-1 blockade. Anti-TGFβ antibodies can break this cycle to reduce TGFβ signaling, reverse abnormal Treg/Th1 cell ratios, and promote tumor rejection and long-term antitumor immunity (Dodagatta-Marri et al. 2019).

### 5.6. Type 17 T Helper Cells: A Pool for Induction of CD4^+^ Type 1 T Helper Cells by TGFβ Inhibition

In a clinical trial for metastatic castration-resistant prostate cancer (mCRPC), Jiao et al. (2019) found that transcriptomic signatures of tumors from two patients, assessed before and after anti-CTLA4 immunotherapy, showed therapy-induced enrichment in CD4^+^ Th1 cells and CD4^+^ Tregs. In contrast, bone marrow samples from mCRPC metastatic sites had few CD4^+^ Th1 cells, but Th17 cell levels were uniformly elevated by anti-CTLA4 therapy in all nine patients examined. It was proposed that osteoclastic release of TGFβ and IL-6 during metastatic growth expands Th17 cells, and this is potentiated by anti-CTLA4 therapy (Yin et al. 1999, Gutcher et al. 2011) (see Figure 3). In a mouse model of mCRPC, combinatorial anti-CTLA4/anti-PD-1 therapy in the subcutaneous setting caused expansion of intratumor effector Th1 cells and TGI, but in the bone metastatic setting, CD4^+^ Th1 cells were depleted and only Th17 cells and CD4^+^ Tregs were quantifiable. Moreover, in metastatic bone marrow, Th17 cells underwent expansion in response to ICB and metastases were resistant to therapy. Notably, the addition of TGFβ blockade to anti-CTLA4 therapy led to an expansion of Th1 cells at the expense of Th17 cells and to the regression of bone metastases (Jiao et al. 2019). It is debatable whether Th17 cells play a positive, negative, or neutral role in tumor progression (Martin et al. 2012, Punt et al. 2016, Asadzadeh et al. 2017), but Jiao et al. (2019) made the point that these TGFβ-induced CD4^+^ Th17 cells might serve as a neutral pool of CD4^+^ T cells from which effector Th1 cells might differentiate under the influence of TGFβ blockade therapy. In a similar manner, Tregs might transition to effector Th1 cells under the influence of TGFβ blockade. Alternatively, Th1 cells may preferentially expand at the expense of Treg and Th17 cell expansion in response to anti-TGFβ therapy (McClymont et al. 2011, D. Wang et al. 2018, Dodagatta-Marri et al. 2019, Martin et al 2020).

### 5.7. Targeting TGFβ Signaling in CD4^+^ Type 2 T Helper Cells Suppresses Tumor Angiogenesis

Protumorigenic activity of TGFβ signaling exerted through effects on CD4^+^ Th2 cells has been reported in theMMTV-PyMT mammary and MC38 colon carcinoma mouse models. Deletion of *Tgfbr2* in mature CD4^+^ T cells using *Thpok-Cre* transgenic mice suppressed tumor outgrowth by relocation of CD4^+^ *Tgfbr2*^−/−^ T cells from their usual site in the tumor parenchyma to the tumor stroma (Liu et al. 2020). This contrasts with the effects of TGFβ inhibition in stimulating CD8^+^ T cell recruitment from the stroma to the tumor parenchyma, which has been observed in several tumor types (Chakravarthy et al. 2018, Mariathasan et al. 2018, Tauriello et al. 2018, Dodagatta-Marri et al. 2019, Gunderson et al. 2020). In the MMTV-PyMT model, *Thpok*-targeted *Tgfbr2* knockout led to the expansion and differentiation of Th2 cells secreting IL-4. This led to IL-4-dependent pericyte investment of the stromal vasculature with reduced tumor angiogenesis. Stromal vascular remodeling was shown to cause distal hypoxia within the tumor parenchyma and resultant cancer cell death (Liu et al. 2020).

To explore the potential of targeting TGFβ signaling blockade specifically to CD4^+^ T cells, researchers developed a novel bispecific therapeutic modality, 4T-Trap, that combines a TGFβR2 ligand trap with an anti-CD4^+^ T cell–binding antibody. Strikingly, in both the MMTV-PyMT and MC38 breast and colon cancer models, 4T-Trap induced cancer hypoxia and cancer cell death, delaying tumor outgrowth but not eliminating tumors. Tumor hypoxia induced by drug treatment enhanced VEGF-A secretion, and coadministration of a VEGF trap and 4T-Trap therapy potentiated tumor cell death and animal survival, although this did not lead to complete tumor rejection (Li et al. 2020).

## 6. TGFβ: EPITHELIAL-MESENCHYMAL TRANSFORMATION, TUMOR STEM CELLS, AND RESISTANCE TO IMMUNOTHERAPY

Many carcinomas, especially those of the gastrointestinal tract have reduced or lost responses to canonical TGFβ signaling through genetic or epigenetic means, including deletion of *TGFBR2, TGFBR1, SMAD2,* or *SMAD4.* In such cases, tumor cells no longer show growth inhibition by TGFβ but secrete cytokines that promote tumor progression, for example, by recruiting and polarizing TAMs, immature myeloid cells, or MDSCs (Kitamura et al. 2007, Yang et al. 2008, Yang & Karin 2014). Paradoxically, this makes these cancers excellent candidates for TGFβ inhibition therapy as it targets TGFβ action within the TME.

In tumors with an intact TGFβR1/2-SMAD2/3 pathway, including most SCCs, glioblastomas, and breast cancers, the activation of oncogenes synergizes with TGFβ signaling to induce EMT rather than TGI. It is increasingly appreciated that the cancer stem cell (CSC) phenotype appears in a transitional state between epithelial and full-blown mesenchymal phenotypes during TGFβ-induced EMT (Dongre & Weinberg 2019, Kroger et al. 2019), and that TGFβ is an established regulator of CSC fate determination and lineage plasticity in normal and malignant tissues (Anido et al. 2010, Calcagno et al. 2010, Connolly et al. 2011, Oshimori & Fuchs 2012, Oshimori et al. 2015, Du et al. 2018, Katsuno et al. 2019, Panda & Biswal 2019).

Importantly, both CSCs and TGFβ signaling have been associated with cancer drug resistance to both chemotherapy and targeted therapies (Huang et al. 2012). TGFβR2 and downstream signaling are enriched in mouse and human CSCs (Shipitsin et al. 2007, Miao et al. 2019), and chronic exposure to TGFβ drives a drug-resistant CSC-like state (Katsuno et al. 2019). Notably, tumor cell TGFβ signaling and EMT also contribute to resistance to immunotherapy. TGFβ suppresses the expression of MHC-I and genes encoding the antigen-processing and -presentation machinery, and TGFβ blockade has been shown to reepithelialize aggressive carcinomas to elevate the expression of MHC-I and antigen presentation and enhance tumor visibility to antigen-specific cytotoxic T cells (Dodagatta-Marri et al. 2019, Lind et al. 2020). TGFβ also upregulates CD80 (B7-1) expression on SCC CSCs. A ligand normally expressed on antigen-presenting cells, CD80 normally binds the T cell costimulatory CD28 receptor, but in the immunosuppressive TME, it binds to the coinhibitory CTLA4 receptor at the T cell–CSC synapse to induce T cell exhaustion (Miao et al. 2019).

CSCs in mouse SCCs are marked not only by TGFβ responsiveness but also by integrin β6 expression, suggesting that activation and signaling of TGFβ occur through this integrin (Miao et al. 2019). Conversely, in human glioblastoma cells, integrin αvβ8 shows a heterogeneous expression that correlates with markers of glioblastoma stem/progenitor cells (Guerrero et al. 2017). Heterotypic cell-cell interactions between malignant cells with high integrin β8 expression and those with low integrin β8 expression result in high TGFβ signaling in the integrin β8–low cells, which is associated with markers of glioblastoma differentiation, and low TGFβ signaling in the integrin β8–high tumor cells, which is associated with the expression of CSC markers, DNA repair, and mitosis. Notably, integrin β8–high glioblastoma cells are better at initiating tumors than are those with low integrin β8 expression, as determined by tumor-sphere formation in vitro and tumor outgrowth in immune-compromised mice in vivo (Guerrero et al. 2017).

Paradoxically, mesenchymal tumors, such as triple-negative breast cancers, appear more responsive to ICB immunotherapy than do epithelial cancers, such as HER2^+^ breast cancer (Nanda et al. 2020). One explanation for this paradox may be that TGFβ-induced EMT causes downregulation of SETDB1 (Du et al. 2018), a chromatin modifier that has been shown to suppress the expression of highly antigenic endogenous retroviral elements that are abundantly scattered throughout mammalian genomes (Griffin et al. 2021). Immunotherapy with a TGFβ signaling blockade agent may activate SETDB1 expression to suppress the expression of these antigenic peptides. Treating tumors with the combination of an anti-TGFβ agent and an SETDB1 inhibitor may potentiate immunotherapy by epithelializing tumors to enhance the antigen-presentation machinery while also activating the expression of antigenic retroviral peptides. The direct action of TGFβ blockade in potentiating immune cell functions would further contribute to tumor rejection.

## 7. DRUGS IN DEVELOPMENT

Many strategies have been taken to drug the TGFβ pathway, including targeting ligands, receptors, or molecules involved in the activation of TGFβ (Figure 1). Drug moieties in clinical development include blocking antibodies, ligand traps, antisense oligonucleotides, and SMIs. In selecting which targets to drug, researchers should take into consideration lessons learned from basic research studies. Blocking TGFβR1 versus TGFβR2 function in CD8^+^ T cells, for example, may lead to different outcomes (compare Liu et al. 2020 and Gunderson et al. 2020), and T cell–targeting of a dominant negative TGFβR2 trap versus deletion of the *TGFBR2* gene in T cells results in different phenotypes (Ishigame et al. 2013a). Moreover, each antibody, including those raised against the same target, has unique properties that may influence antitumor efficacy.

Although several antibodies that block TGFβ ligands are under clinical investigation (Table 1) and an anti-TGFβR2 receptor been clinically tested, the drugs most widely tested in the clinic have been SMIs of TGFβR1 kinase, such as galunisertib and vactosertib. SMIs suffer from lack of specificity and from a small window between therapeutic response and potential cardiotoxicity (as seen in animals at very high doses). Cardiotoxicity is also seen at high doses of potent nonclinical anti-ligand antibodies (Mitra et al. 2020). Future drug development should therefore aim toward next-generation SMIs that target other components of the TGFβ signaling pathway, particularly molecules whose expression is restricted primarily to tumors, or that target TGFβ blockade for specific cell types (Dodagatta-Marri et al. 2021), including via incorporation into CAR T cells or oncolytic viruses, etc. (Hou et al. 2018, Groeneveldt et al. 2020).

Bintrafusp alfa is a bispecific protein that combines an anti-PD-L1 antibody, based on avelumab, with a TGFβ1/3 ligand trap, based on the extracellular domain of TGFβR2 (Jochems et al. 2017). The concept behind bintrafusp alfa is that the anti-PD-L1 moiety targets drug delivery to the tumor, concentrating TGFβ depletion (and anti-PD-L1 activity) to sites of high PD-L1 in order to decrease any adverse side effects related to TGFβ blockade in normal tissue. In vivo positron emission tomography imaging of the radiolabeled drug shows preferential accumulation within tumors, with some accumulation in the kidney (Burvenich et al. 2021). Nevertheless, bintrafusp alfa was shown to deplete all circulating TGFβ (Lan et al. 2018), which suggests a systemic effect, and a finding of hemorrhaging from mucosal surfaces during therapy, albeit clinically manageable, also suggests systemic TGFβ blockade since this was not observed with anti-PD-L1 monotherapy (Strauss et al. 2018). Bintrafusp alfa mediates tumor regression and long-term antitumor immunity in rodent experiments (Knudson et al. 2018, Lan et al. 2018), and in phase I and II clinical trials it showed enhanced efficacy compared to anti-PD-L1 monotherapy, particularly in human papilloma–positive tumors (Strauss et al. 2018, Strauss et al. 2020). The drug increased intratumoral Th1 cell/Treg and CD8^+^ T cell/Treg ratios, activated NK cells, elevated intratumoral monocytes at the expense of MDSCs, induced cytotoxic NK cell properties in CD4^+^ and CD8^+^ T cells, and reverted mesenchymal tumors toward an epithelial phenotype, all of which are features of TGFβ inhibition not observed with anti-PD-L1 monotherapy.

Recently, two late-stage clinical trials of bintrafusp alfa failed to reach their predefined threshold for clinical success, but the threshold was set high. In a trial of newly diagnosed late-stage NSCLC bintrafusp alfa did not sufficiently outperform Keytruda^®^ (an anti-PD-1 therapeutic) (Adams 2021). In a second halted trial of 159 patients with locally advanced or metastatic biliary tract cancer (BTC) who had failed other therapies, bintrafusp alfa monotherapy provided a 10.1% objective response rate (ORR) (Adams 2021). Placing these data into context, bintrafusp alfa compared favorably with current standard therapy for metastatic BTC, namely an anti-PD-L1 that shows a historical ORR of only 5.8% in all patients (Adams 2021), including those with no prior drug treatment who are generally less resistant to therapy. However, the 10% ORR achieved with bintrafusp alfa did not pass the predefined threshold for clinical success. Nevertheless, bintrafusp alfa continues to be developed for other cancers and in combination with other drugs, such as chemotherapy (Adams 2021).

The drug design of bintrafusp alfa is elegant, and preclinical studies have shown that combining the two biological activities within one molecule did not compromise the efficiency of either. However, the molecular constraints imposed by restricting TGFβ blockade to sites of PD-L1 within the tumor may have compromised the efficacy of the neutralization of active TGFβ. This is pertinent, considering the intimate molecular relationship between activation of latent TGFβ by integrin β8 and the initiation of TGFβ/TGFβR signaling. It is possible that the combination of a TGFβ inhibitor with an independently administered drug targeting the PD-1/PD-L1 axis, which is being trialed by many pharmaceutical companies, may be more efficacious and provide the ability to titrate optimal drug dosing for each component independently.

## 8. TARGETING ACTIVATION OF TGFβ FOR CANCER IMMUNOTHERAPY

It is increasingly clear that targeting activation of TGFβ is a highly attractive approach to stimulate ICB therapy, and recent studies suggest that integrin β8 is an excellent target. In adults, integrin β8 expression is mainly confined to T cells, macrophages, and DCs, and its expression is upregulated in tumors where it is expressed on malignant cells and CD4^+^ T cells in a slew of mouse and human cancer types (Reyes et al. 2013, Guerrero et al. 2017, Takasaka et al. 2018, Dodagatta-Marri et al. 2021, Seed et al. 2021). Importantly, in colon carcinoma, triple-negative basal-type breast cancer, advanced-stage serous ovarian cancer, and NSCLC, high integrin β8 expression is associated with poor clinical outcomes (Takasaka et al. 2018, Zhou et al. 2020).

Structural and empirical studies have shown that the activation of latent TGFβ by integrin β8, which occurs concomitantly with the engagement of mature TGFβ with TGFβR2, occurs within a geometrically constrained complex formed between αvβ8 on one cell interacting with latent TGFβ presented on another (Figure 2b). TGFβ signaling can therefore occur without the need for the release and diffusion of active TGFβ. The molecular constraints imposed within this complex were predicted to diminish access to anti-TGFβ-blocking antibodies or ligand traps (Campbell et al. 2020, Seed et al. 2021). These observations provide credence to the view that targeting integrin αvβ8 activation of latent TGFβ with specific anti–integrin β8 antibodies (Takasaka et al. 2018, Dodagatta-Marri et al. 2021) or small molecules (Reichart et al. 2019) may be more efficacious than blocking the free ligand and less likely to instigate systemic adverse effects away from the tumor site.

Integrin β8 (Worthington et al. 2015) and GARP (Stockis et al. 2009, Cuende et al. 2015) are both expressed on activated human and mouse CD4^+^ FOXP3^+^ Tregs, and αvβ8 expression is elevated on Tregs during inflammatory activation (Worthington et al. 2015), including within tumors compared to normal lymphoid tissues (Dodagatta-Marri et al. 2021). Genetic deletion of *Itgb8* in Tregs using a FOXP3-Cre-driven mouse did not break Treg-mediated tolerance under homeostatic conditions but did block the suppression of T cell–mediated inflammation (Worthington et al. 2015), which is important for considering systemic integrin β8 inhibition for therapy. Recent studies have demonstrated remarkable tumor regression and antitumor immunity in response to antibody blockade of αvβ8. In multiple mouse tumor models, including SCCs and mammary, prostate, and lung cancers that express a range of cell surface integrin β8 levels, anti-integrin β8 antibodies, even as monotherapy, exerted efficient antitumor responses, including downregulation of intratumoral pSMAD2/3 signaling, tumor rejection, and long-term anti-tumor immunity (Takasaka et al. 2018, Dodagatta-Marri et al. 2021, Laine et al. 2021). In some cancer models, this was potentiated by ICB therapies, including anti-PD-1 therapy with or without radiation, anti-CTLA4 therapy, or a 4-1BB agonist (Dodagatta-Marri et al. 2021). Within the tumor, the highest *Itgb8* expression levels were observed in CD4^+^ T cells, particularly in CD4^+^CD25^+^ Tregs compared to conventional CD4^+^CD25^−^ T cells. *Itgb8* RNA was much lower in tumor cells and other lymphocytic and myeloid cell types. Importantly, *Itgb8* expression in intratumoral Tregs was elevated three- to sixfold compared to that in Tregs of other lymphoid tissue, and deletion of *Itgb8* in T cells using a *Cd4-Cre* transgene in a transplantable syngeneic prostate tumor model was as effective as, and redundant with, administration of anti–integrin β8 antibodies in delaying tumor outgrowth and extending life (Dodagatta-Marri et al. 2021). In contrast, deletion of *Itgb8* in DCs using a *Cd11c-Cre* promoter had no effect on tumor growth, confirming the important role of T cell–specific *Itgb8* expression in driving tumor growth via TGFβ activation (Dodagatta-Marri et al. 2021). In a separate study, *Itgb8* expression was deleted in CD4^+^ Tregs using a *Foxp3-Cre* transgene, leading to dramatic reduction in outgrowth of implanted E0117 mammary tumor cells, validating the concept that activation of TGFβ by integrin β8 expressed on Tregs is a critical component of the TGFβ-mediated immunosuppressive machinery in the tumor (Laine et al. 2021). A third study did not detect integrin β8 on intratumoral mouse or human Tregs but showed high integrin β8 protein expression on the surface of human and mouse tumor cells (Seed et al. 2021). Using tumor lines expressing different integrin β8 levels, and by manipulating tumor cell *Itgb8* expression genetically, the researchers found that Treg infiltration in vivo correlates with tumor cell expression of integrin β8, inferring that integrin β8 on tumor cells leads to TGFβ-induced Treg differentiation that contributed to immune-excluded tumors (Seed et al. 2021). Integrin β8 expression on tumor cells has also been shown to play a role in supporting tumor growth independent of any effect on immune cells, as demonstrated in mouse and human glioblastomas (Guerrero et al. 2017).

Antibodies have also been developed that stabilize TGFβ latency by binding the RGD site of LAPs to compete with integrins, and these have been found to be efficacious in preclinical models (Martin et al. 2020). However, such anti-LAP antibodies may target the activation of TGFβ more widely rather than focal tumor–specific effects of integrin β8 blockade. Proof of concept for all these drugs awaits the outcome of currently ongoing clinical trials.

## 9. CONCLUSIONS AND FUTURE DIRECTIONS

In conclusion, markers of EMT, ECM, activated CAFs, and high-TGFβ signaling are prognostic features associated with a lack of tumor responses to ICB therapy, with the first three of these potentially driven at least in part by TGFβ. Intratumoral TGFβ signaling is elevated further by ICB and radiation therapy or chemotherapy. It is therefore not surprising that in many preclinical models, drugging the TGFβ signaling pathway has been shown to synergize with ICB therapy through diverse mechanisms dependent on the tumor type and grade. TGFβ blockade relieves the immunosuppression of cytotoxic CD8^+^ T cells and NK cells, promotes lineage switching within the CD4^+^ T cell population, depolarizes immunosuppressive intratumoral myeloid cells and CAFs, and inhibits angiogenesis, leading to tumor regression and long-term immunity. Drugs in clinical development include antibodies that block active ligands, block TGFβ-activating integrins, or stabilize latent TGFβ; ligand traps; antisense ligands; and bispecific ligand traps that target the TGFβ blockade to specific sites/cells or that have ICB moieties. Targeting the activation of latent TGFβ through the blockade of integrin β8 may increase efficacy and reduce adverse effects because the drug target has more restricted expression. However, there is room for further improvements in drug design, drug dosing regimens, and patient stratification for TGFβ blockade agents.

On the basic research side, areas for future investigation might include the impact of tumor exosomes on TGFβ blockade therapies and vice versa. Tumor-derived exosomes or so-called apoptotic bodies have protumorigenic and prometastatic properties (Hoshino et al. 2015, Becker et al. 2016) and have been shown to package and present both TGFβ (Xie et al. 2009, Wada et al. 2010, Webber et al. 2010) and PD-L1 (Poggio et al. 2019) to immune and malignant cells to drive tumor progression. In fact, TGFβ has been shown to be instrumental in orchestrating the enrichment of PD-L1 in exosomes to suppress CD8^+^ T cells in breast cancer (Chatterjee et al. 2021). Consideration of these issues will be important for drug design since, for example, exosomal PD-L1, which drives tumor growth, has been shown to be resistant to systemic anti-PD-L1 therapy in a prostate cancer model (Poggio et al. 2019). Development of nanobodies against TGFβ signaling targets should give better access to drug targets than conventional antibodies (Chanier & Chames 2019). Moreover, novel molecular targets on the TGFβ signaling pathway may still be identified that increase the window between therapeutic response and adverse effects.

Most importantly, since each tumor type utilizes TGFβ signaling to drive a different aspect of tumor progression, a major challenge is to identify the overriding cellular targets and molecular mechanisms that drive TGFβ blockade responses for each tumor class. Additionally, specific tumor-driving mutations or genomic rearrangements may influence the efficacy of TGFβ signaling blockade immunotherapy. In this respect, it will be important to develop high-throughput prognostic biomarkers that are predictive of outcomes of TGFβ signaling blockade therapies to allow for patient stratification and to develop robust pharmacodynamic markers for the longitudinal assessment of therapeutic responses versus progressive disease or the development of adverse effects.

## Figures and Tables

**Table 1:** Therapeutic strategies in clinical oncology development for TGFβ signaling blockade

Approach	Target(s)	Drug	Cancer type(s)	Clinical phase	Clinical trial number or reference
Antibody	Blocking pan-TGFβ (TGFβ1, TGFβ2, and/or TGFβ3)	Fresolimumab (pan-TGFβ)	Relapsed pleural malignant mesothelioma	II	NCT01112293
			With radiotherapy in metastatic breast cancer	II	NCT01401062
			Renal cell carcinoma, melanoma, and glioma	II	NCT00356460, NCT00923169, NCT01472731
			With targeted ablation radiotherapy for NSCLC	II	NCT02581787
		SAR439459	With cemiplimab (anti-PD-1) for advanced-stage or unresectable solid tumors	I/Ib	NCT03192345, NCT04729725
		NIS793	In combination with anti-PD-1 for advanced malignancies	I/Ib	NCT02947165
			With bevacizumab, FOLFOX6, or FOLFIRI for second-line therapy of metastatic colon cancer	II	NCT04952753
			In combination with SOC chemotherapy for first-line treatment of metastatic ductal pancreatic cancer	III	NCT04935359
			In combination with SOC chemotherapy with/without spartalizumab (anti-PD-1) for first-line treatment of metastatic ductal pancreatic cancer	III	NCT04390763
	GARP: TGFβ1	ABBV-151	With/without budigalimab (anti-PD-1) for locally advanced or metastatic solid tumors	II	NCT03821935
	LAP: TGFβ1	SRK-181	Locally advanced or metastatic solid tumors	I/Ib	NCT04291079
	Integrin αvβ8	PF06940434	Advanced or metastatic solid tumor malignancies with/without anti-PD-1	I/Ib	NCT04152018
Ligand trap	TGFβ1 and -3	AVID200	Advanced and metastatic cancers, recurrent/refractory NSCLC, HCC, rectal cancer, ovarian carcinosarcoma, and newly diagnosed malignant glioma	I and II	NCT03834662
Small-molecule inhibitor	TGFβR1	Galunisertib	Advanced and metastatic cancers, multiple myeloma, urothelial carcinoma, and myeloproliferative neoplasm	I and II	NCT02906397, NCT02452008, NCT02688712, NCT02423343, NCT02734160, NCT01220271, NCT01246986, NCT02672475, NCT03206177, NCT01373164, NCT01373164
		Vactosertib	Colorectal cancer and solid tumor	I and II	NCT04064190, NCT03143985, NCT03724851, NCT04103645, NCT03802084, NCT03732274, NCT03698825, NCT04258072, NCT02160106
		LY3200882	Solid tumors	I	NCT04031872, NCT02937272, NCT02937272
		PF-06952229	Advanced NSCLC, advanced/metastatic/recurrent cancers, glioblastoma, brain metastasis from lung cancer, and glioma	I, II, and III	NCT03685591
Bispecific molecule	TGFβ1 and -3 plus PD-L1	Bintrafusp alfa	Various; particularly for HPV^+^ cancers	I, II, and III	Multiple ongoing trials, although two trials terminated due to lack of phase III efficacy compared to competitors

Abbreviations: FOLFIRI, folinic acid, fluorouracil, and irinotecan; FOLFOX6, folinic acid, fluorouracil, and oxaliplatin; GARP, glycoprotein A repetitions predominant; HCC, hepatocellular carcinoma; HPV, human papilloma virus; LAP, latency-associated peptide; NSCLC, non-small-cell lung cancer; SOC, standard of care.

## IMMUNE CHECKPOINT BLOCKADE

ICB agents act by preventing T cell exhaustion. Therapeutic antibodies that block ligation of PD-1 on T lymphocytes with its tissue-localized ligands, PD-L1 and PD-L2, have revolutionized cancer therapy particularly for high-TML (tumor mutation load) tumors with high neoantigen content. Long-term durable responses, including complete regressions, are often seen, although only a minority of patients benefit without the use additional drugs in combination with anti-PD-1/PD-L1. The first FDA (US Food and Drug Administration)-approved ICB agent prevented ligation of T cell–presented CTLA4 to its ligands that are expressed on antigen-presenting cells. Many other ICB agents are in drug development.

## TGFβ ACTIVITIES IN HEALTH AND DISEASE

TGFβ was discovered in the late 1970s to early 1980s as a component of a secreted factor (now known to be TGFα and TGFβ) that could transform fibroblast cells, allowing them to grow in soft agar. It was soon found that TGFβ is a potent inhibitor of normal epithelial proliferation, but efforts to harness this activity to prevent tumor outgrowth were thwarted by the development of tumor cell insensitivity to TGFβ-mediated growth arrest. This cytokine is a pleiotropic signaling molecule that plays important and highly regulated roles during development, differentiation, and tissue homeostasis. It is transiently induced upon the perturbation of homeostasis, such as in wounding, inflammation, or tumor promotion, but in pathological situations, including cancer and fibrosis, TGFβ secretion is chronically elevated and contributes to the exacerbation of the diseased state. Excessive amounts of TGFβ produced from multiple cellular sources within the tumor act locally on both malignant cells and cells of the tumor microenvironment (TME) to support tumor progression and metastasis.

## INTEGRINS

Integrins are heterodimeric transmembrane proteins possessing an α and a β subunit. They bind RGD motifs within ECM proteins, such as fibronectin, and integrate communication between the ECM and intracellular cytoskeleton. Integrins transduce signals from ECM to cytoskeleton and from cytoskeleton to ECM, termed outside-in and inside-out signaling (Hynes 2002, Cooper & Giancotti 2019, Michael & Parsons 2020). Generally, unligated integrin takes on a folded inactive state. Integrin activation or inside-out signaling is initiated by cytoplasmic proteins like talin, which bind to the intracellular tail of the integrin β subunit, causing separation of the α and β chains on the cytosolic side and conformational change that causes extension of the extracellular domain and results in increased integrin affinity for its extracellular ligands.

## TGFβ:

transforming growth factor β

## ICB:

immune checkpoint blockade

## EMT:

epithelial-mesenchymal transformation

## ECM:

extracellular matrix

## MDSCs:

myeloid-derived suppressor cells

## CAFs:

cancer-associated fibroblasts

## TAMs:

tumor-associated macrophages

## LAP:

latency-associated peptide

## LTBP:

latent TGFβ-binding protein

## GARP:

glycoprotein A repetitions predominant

## NRROS:

negative regulator of reactive oxygen species

## TML:

tumor mutation load

## SCCs:

squamous cell carcinomas

## NK:

natural killer

## MHC:

major histocompatibility complex

## NSCLC:

non-small-cell lung cancer

## TGI:

tumor growth inhibition

## NSG-β2m^−/−^:

gene knockout of the *B2m* gene on a highly immunodeficient *NOD* SCID *Il2rg*^*−/−*^ mouse
